# Dalron on the Constitution and Physiology of Bile

**Published:** 1857-11

**Authors:** 


### EDITORIAL DEPARTMENT.

---

*Dalton, on the Constitution and Physiology of Bile.*—A careful perusal of the paper which occupies the bulk of our Eclectic Department will convince our readers that it could be occupied by no more interesting matter. Dr. Dalton's name as Prof. of Physiology, etc., in the Buffalo University, is sufficiently well known to all, and the reputation which he has acquired since his connexion with that institution, will be materially increased by this paper, which is the result of two years' constant investigation. We make this allusion because elaborate papers on subjects not immediately practical are apt to be passed over as too abstruse for ordinary readers. The paper by Dr. Dalton requires, and we hope it will receive a careful study. We had hoped to obtain the woodcuts by which it was illustrated in the American Journal, but were unable to do so; they would of course have added to the interest of the article though they are by no means indispensible. F.

